# CARMA2*sh* and ULK2 control pathogen-associated molecular patterns recognition in human keratinocytes: psoriasis-linked CARMA2*sh* mutants escape ULK2 censorship

**DOI:** 10.1038/cddis.2017.51

**Published:** 2017-02-23

**Authors:** Ivan Scudiero, Pellegrino Mazzone, Luca E D'Andrea, Angela Ferravante, Tiziana Zotti, Gianluca Telesio, Gabriele De Rubis, Carla Reale, Maddalena Pizzulo, Shanmugakonar Muralitharan, Pasquale Vito, Romania Stilo

**Affiliations:** 1 Biogem, Via Camporeale, Ariano Irpino, Italy; 2 Genus Biotechnology, Universita' del Sannio, Via Port'Arsa 10, Benevento, Italy; 3 Laboratory Animal Research Centre, Qatar University, PO Box 2713, Doha, Qatar; 4 Dipartimento di Scienze e Tecnologie, Università del Sannio, Via Port'Arsa 10, Benevento, Italy

## Abstract

The molecular complexes formed by specific members of the family of CARMA proteins, the CARD domain-containing adapter molecule BCL10 and MALT1 (CBM complex) represent a central hub in regulating activation of the pleiotropic transcription factor NF-*κ*B. Recently, missense mutations in CARMA2*sh* have been shown to cause psoriasis in a dominant manner and with high penetrancy. Here, we demonstrate that in human keratinocytes CARMA2*sh* plays an essential role in the signal transduction pathway that connects pathogen-associated molecular patterns recognition to NF-*κ*B activation. We also find that the serine/threonine kinase ULK2 binds to and phosphorylates CARMA2*sh*, thereby inhibiting its capacity to activate NF-*κ*B by promoting lysosomal degradation of BCL10, which is essential for CARMA2*sh*-mediated NF-*κ*B signaling. Remarkably, CARMA2*sh* mutants associated with psoriasis escape ULK2 inhibition. Finally, we show that a peptide blocking CARD-mediated BCL10 interactions reduces the capacity of psoriasis-linked CARMA2*sh* mutants to activate NF-*κ*B. Our work elucidates a fundamental signaling mechanism operating in human keratinocytes and opens to novel potential tools for the therapeutical treatment of human skin disorders.

Psoriasis is a debilitating skin disease affecting ~2–3% of human population.^1^ The disease is considered to have key genetic underpinnings, and genome-wide association studies and meta-analysis have identified more than 40 susceptibility loci for psoriasis.^1, 2, 3, 4, 5^ Recently, missense mutations in the *CARD14/CARMA2/Bimp2* (*CARMA2*) gene products have been shown to dominantly transmit the psoriatic trait with high penetrancy.^6, 7, 8^ Mutations in *CARMA2* are also found in individuals affected by familial pityriasis rubra pilaris (PRP), a skin disorder phenotypically related to psoriasis.^9^ When introduced in CARMA2*sh*, the prominent CARMA2 isoform expressed in the human skin,^7, 10, 11^ most CARMA2 mutations associated with psoriasis and PRP cause a deregulated, enhanced activity of NF-*κ*B, a transcription factor that catalyzes expression of inflammatory genes.^6, 7, 9, 10^ Indeed, similarly to classic psoriasis, transcriptomes of psoriatic patients carrying mutations in *CARMA2* are significantly enriched in transcripts functioning in inflammatory pathways including the LPS/IL-1 pathways.^10^ CARMA2*sh* activates NF-*κ*B through assembly of the CARMA/BCL10/MALT1 (CBM) complex, a molecular complex that in addition to CARMA2*sh* also includes the paracaspase MALT1 and the adapter molecule BCL10.^11, 12, 13^ BCL10 is a critical mediator of NF-*κ*B activation in immune cells, and genetic deficiency of BCL10 results in combined immunodeficiency in both humans and mice, due to impaired NF-*κ*B activation following antigen receptor stimulation.^14, 15, 16^ In addition, in lymphoid cells selective degradation of BCL10 represents an homeostatic mechanism that prevents unrestrained NF-*κ*B signaling.^17, 18, 19^ More recent reports have also identified BCL10 as a positive regulator of inflammatory cascades in non-immune cells, including the LPS-toll-like receptor (TLR)4-induced pathway of NF-*κ*B activation.^20, 21, 22^ Here, we find that the serine/threonine kinase ULK2 binds to and phosphorylates CARMA2*sh*, thereby inhibiting its capacity to activate NF-*κ*B by promoting lysosomal degradation of BCL10. Remarkably, CARMA2*sh* mutants associated with psoriasis escape ULK2 inhibition. We also show that in human keratinocytes the CBM complex plays an essential role in the signal transduction pathway that links pathogen-associated molecular patterns (PAMPs) recognition on the cell membrane to NF-*κ*B activation.

## Results

### ULK2 binds to and phosphorylates CARMA2sh

Most of the studies conducted to elucidate the functional role of CARMA2 and its mutated forms associated with psoriasis have been carried out using CARMA2*sh*,^6, 7, 8, 9, 11^ the prominent isoform of CARMA2 expressed in the human skin.^7, 10^ Thus, to search for functional regulators of CARMA2*sh* we performed a yeast two-hybrid screen using as a bait CARMA2*sh* fused to the GAL4 DNA-binding domain. A total of 14 yeast clones were isolated that activated the *β*-galactosidase reporter gene when ~2 × 10^6^ library plasmids were screened. Sequence analysis of these CARMA2*sh* putative interactors revealed that one isolated plasmid encoded for amino acids Leu^805^–Val^1036^ of ULK2, a serine/threonine kinase involved in autophagy.^23, 24^ As summarized in Table 1, this library clone did not activate the yeast GAL4-reporter genes by itself or when co-expressed with the empty GAL4BD vector, or with a control plasmid. Conversely, it strongly interacted with GAL4BD-CARMA2*sh* fusion protein.

**Figure 1:**
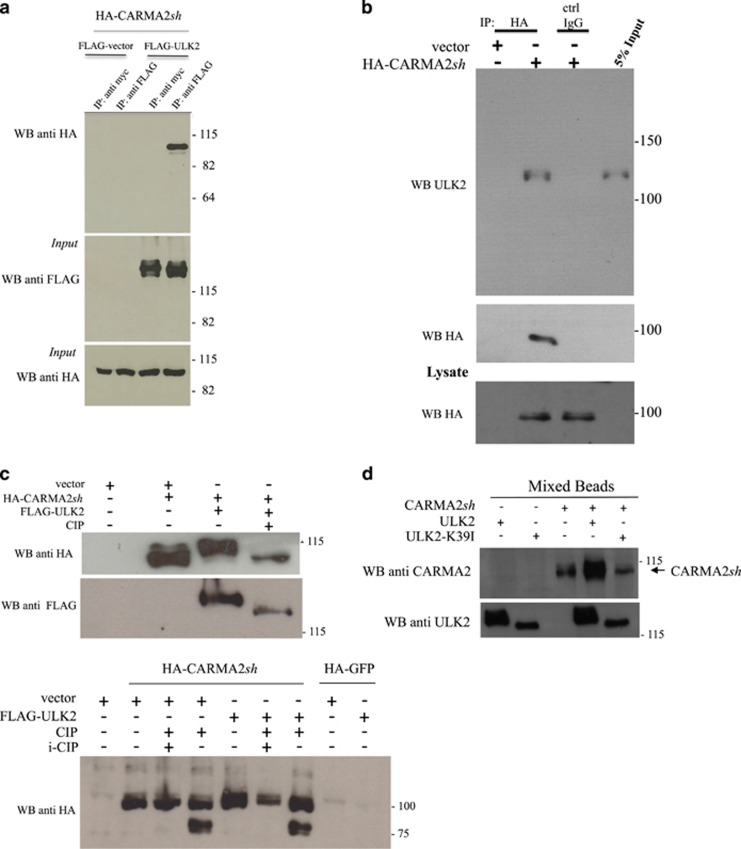
ULK2 binds to and phosphorylates CARMA2*sh*. (**a**) HEK293T cells were cotransfected with a plasmid encoding for HA-tagged CARMA2*sh* together with a FLAG-tagged expression vector empty or encoding for ULK2. About 24 h later, lysates were immunoprecipitated with anti-FLAG or anti-myc control antibodies and analyzed for coprecipitating HA-CARMA*sh* by western blot assay. (**b**) HaCaT cells were transfected with either an empty plasmid or encoding for HA-tagged CARMA2*sh.* About 24 h later, cells were lysed and immunoprecipitated with anti-HA or anti-myc control antibodies, and analyzed for coprecipitating endogenous ULK2 protein by western blot assay. (**c**) HEK293T cells were cotransfected with a plasmid encoding for HA-tagged CARMA2*sh* together with an expression vector encoding for ULK2. About 24 h later, CARMA2*sh* expression was analyzed by immunoblot assay probed with anti-HA. Where indicated, the cell lysate was treated with CIP (0.5 U/*μ*g lysate) for 30 min at 37 °C and/or a mixture of phosphatase inhibitors (i-CIP). (**d**) Lysates from HEK293T separately transfected with the indicated expression plasmids were immunoprecipitated and tested in a mixed beads *in vitro* kinase assay as described in Material and Methods section. The slower migration in SDS/PAGE of wt ULK2 compared to ULK2K39I is due to ULK2 autophosphorylation (data not shown)

Interaction between CARMA2*sh* and ULK2 also occurs in mammalian cells, as FLAG-tagged full-length ULK2 coprecipitates with CARMA2*sh* when lysates prepared from cotransfected HEK293T cells were immunoprecipitated with an anti-FLAG antibody, but not with an anti-myc control antibody (Figure 1a). Also, transfected CARMA2*sh* coprecipitates endogenous ULK2 in the human keratinocyte HaCaT cell line (Figure 1b).

In performing these co-expression experiments, we observed that in SDS-PAGE separations CARMA2*sh* migrates as a doublet when expressed together with ULK2 in HEK293T cells, suggesting that ULK2 could promote phosphorylation of CARMA2*sh* (Figure 1c, upper and lower panels). In fact, CARMA2*sh* doublets disappear when lysates prepared from cotransfected HEK293T cells were treated with calf intestinal phosphatase (CIP) for 30 min immediately before SDS-PAGE separation (Figure 1c, upper panel). For instance, transfected CARMA2*sh* in SDS/PAGE migrates at an higher molecular mass than that predicted from its amino acid sequence (83.6 kDa predicted *versus*≈100 kDa observed in SDS/PAGE) (Figure 1c, lower panel, lane 2). When lysates were treated with CIP, a signal appears at ≈80 KDa (lane 4), which corresponds to the predicted molecular mass of CARMA2*sh*. The ≈80 KDa band disappears again when lysates were treated with a mixture of phosphates inhibitors (lanes 3 and 6), confirming that the ≈100 KDa signal includes phosphorylated forms of CARMA2*sh.* Of note, co-transfection of ULK2 with CARMA2*sh* further increases the molecular mass shift of CARMA2*sh* (lane 5). Overall, these results demonstrate that a consistent fraction of transfected CARMA2*sh* is present in the cells in a phosphorylated form, and that ULK2 is able to phosphorylate CARMA2*sh*. Moreover, as shown later in Supplementary Figure 3, CARMA2*sh* doublets were not observed when CARMA2*sh* was expressed together with the kinase inactive mutant ULK2K39I.^21^ Finally, in a mixed beads *in vitro* kinase assays wt ULK2, but not ULK2K39I, promoted a shift in the SDS/PAGE mobility of CARMA2*sh* (Figure 1d). Considering altogether these results, we concluded that ULK2 promotes, directly or indirectly, phosphorylation of CARMA2*sh*.

Next, we tried to map the region of CARMA2*sh* phosphorylated by ULK2 using deletion mutants of CARMA2*sh*. The results of these experiments, shown in Supplementary Figure 1, mapped the phosphorylation site of CARMA2*sh* in the linker region of the protein, within the amino acidic stretch Asp^458^–Ser^500^, which contains 10 serine residues and 1 threonine residue. Indeed, a construct encoding for a deleted version of CARMA2*sh* lacking this region (CARMA2*sh*Δ458-500; predicted molecular mass 79.9 KDa), that we generated and used later in this work, migrates at the expected position in SDS/PAGE separations (Supplementary Figures 3 and 4).

### ULK2 represses the NF-*κ*B-inducing activity of wt CARMA2sh, but not that of the non-phosphorylatable or psoriasis-linked CARMA2sh mutants

**Figure 2:**
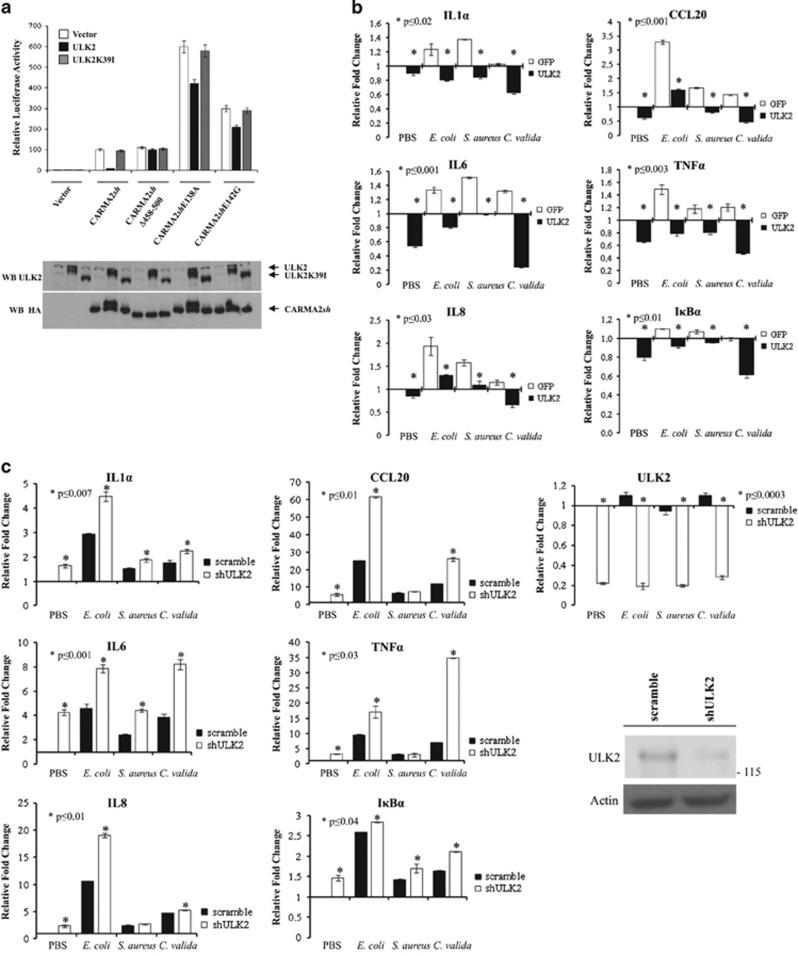

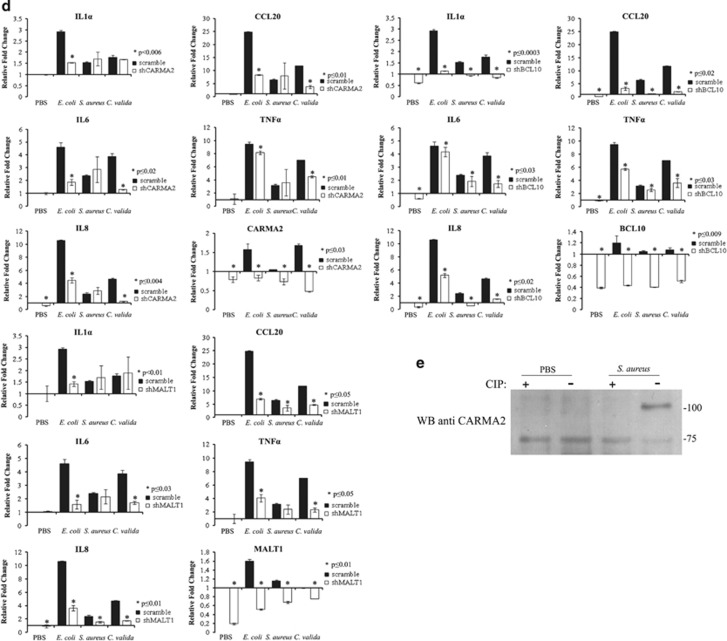
ULK2 represses the NF-*κ*B-inducing activity of CARMA2*sh*. (**a**) HEK293T cells were transiently cotransfected with expression vectors encoding for the indicated polypeptides, together with NF-*κ*B-luciferase and *β*-galactosidase reporter vectors. About 24 h after transfection, cell lysates were prepared and luciferase activity was measured. Data were analyzed by Student's *t*-test, and a *P*-value ⩽0.05, indicated with an *, was considered significant. Data shown represent relative luciferase activity normalized against *β*-galactosidase activity and are representative of at least 10 independent experiments done in triplicate. Lower panel: a fraction of the cell lysate was analyzed by immunoblot assay to monitor protein expression. (**b**) NHEK were transfected with an expression vector encoding for ULK2 or GFP. About 24 h later, cells were left in PBS or exposed to the indicated heat-killed microorganisms for 16 h, and the expression level of selected NF-*κ*B target genes was monitored by real-time PCR. Graphs show the fold changes respect to the GFP-transfected cells left in PBS. Data were analyzed by Student's *t*-test, and a *P*-value ⩽0.05, indicated with an *, was considered significant. Data shown are representative of at least three independent experiments done in triplicate. (**c**) NHEK cells were infected with lentiviral vectors encoding for shULK2 or a scramble control sequence, and then treated and assayed as in **b**. Graphs show the fold changes respect to the scramble-infected cells left in PBS. Data shown are representative of at least three independent experiments done in triplicate. Efficacy of ULK2 attenuation by shRNAs was monitored by real-time PCR and immunoblot assay. (**d**) NHEK were infected with lentiviral vectors encoding for indicated shRNAs or a scramble control sequence and then treated and assayed as in (**c**). (**e**) NHEK cells were left in PBS or exposed to heat-killed *S. aureus* cells for 16 h. Where indicated, the cell lysate was treated with CIP for 30 min at 37 °C and endogenous CARMA2*sh* expression was analyzed by immunoblot assay

Most of the psoriasis-linked CARMA2*sh* mutants enhance the transcriptional activity of NF-*κ*B,^6, 7, 8, 9, 11^ which catalyzes transcription of inflammatory mediators and plays a significant role in the progression of the disease.^25^ Thus, we tested the effect of ULK2 on the NF-*κ*B-inducing activity of CARMA2*sh*. Preliminary experiments carried out using an NF-*κ*B-luciferase reporter assay and shown in Figure 2a, indicated that wt ULK2, but not ULK2K39I, dramatically diminishes the NF-*κ*B-inducing activity of CARMA2*sh*.

Importantly, in the same assay, the NF-*κ*B-inducing activity of the non-phosphorylatable mutant CARMA2*sh*Δ458-500 was not affected by ULK2 expression, suggesting that CARMA2*sh* phosphorylation is necessary for ULK2 to operate its negative control on the NF-*κ*B-inducing activity of CARMA2*sh*. Even more importantly, two of the most recurrent psoriasis-linked mutants, CARMA2*sh*E138A and CARMA2*sh*E142G, which are both stronger activators of NF-*κ*B compared to wt CARMA2*sh*,^6, 7, 8^ were only slightly affected by ULK2 expression.

### ULK2 represses expression of inflammatory genes in human keratinocytes exposed to PAMPs

Subsequently, we investigated the negative effect of ULK2 on the NF-*κ*B-inducing activity of CARMA2*sh* in a more physiological experimental system. Given the evidence that CARMA2*sh* is predominantly expressed in the human skin,^7, 10^ we decided to use normal human epidermal keratinocytes (NHEK) for a panel of experiments aimed at defining a physiological NF-*κ*B activation pathway dependent on CARMA2*sh* and inhibited by ULK2. At this point, however, we faced a significant issue. In fact, although it is known that CARMA1 intervenes in the signal transduction pathway that leads to NF-*κ*B activation in B and T lymphocytes following antigen receptor stimulation,^12, 13, 14, 15^ and that CARMA3 controls NF-*κ*B activation following G-coupled protein receptors triggering in a variety of cell types,^12, 13, 14, 15^ it is currently not known from which membrane receptor(s) signaling, if any, CARMA2*sh* triggers NF-*κ*B activation. While testing a number of different stimuli, including several inflammatory cytokines such as TNF*α* and IL-1*β*, it has not escaped our notice that a significant repression of inflammatory NF-*κ*B target genes was observed when NHEK-expressing ULK2 were exposed to heat-killed bacterial (*Escherichia* *coli* and *Staphylococcus* *aureus*) or fungi (*Chorizanthe* *valida*) cells (Figure 2b). Heat-killed bacterial and fungi cells display PAMPs and activate NF-*κ*B upon agonistic binding to pattern recognition receptors, including members of the TLRs family expressed on human keratinocytes.^26, 27^ Consistently with the experiments shown in Figure 2a, short interfering (sh) RNA targeting ULK2 enhanced the expression level of these inflammatory NF-*κ*B target genes in NHEK exposed to the same stimuli (Figure 2c). Thus, in human keratinocytes ULK2 regulates NF-*κ*B activation ensuing from PAMPs recognition, possibly acting on the CBM complex-mediated signaling.

### The CARMA2/BCL10/MALT1 complex connects PAMPs recognition to NF-*κ*B activation

The inhibitory effect on NF-*κ*B activation exerted by ULK2 in NHEK exposed to PAMPs could be due either to an ULK2-mediated suppression of the NF-*κ*B-inducing activity of CARMA2*sh*, or to an ULK2-sensitive but CARMA2*sh*-independent mechanisms. To test for this, we used shRNA to individually deplete NHEK of the CBM complex components, id est CARMA2, BCL10 and MALT1. As shown in Figure 2d, abrogation of either CARMA2*sh* or BCL10 or MALT1 significantly impairs expression of NF-*κ*B target genes in NHEK exposed to PAMPs. Remarkably, NHEK exposure to heat-killed *S. aureus* promotes a shift in the SDS-PAGE mobility of endogenous CARMA2sh, which was abolished by phosphatase treatment (Figure 2e). These data clearly indicate that in human keratinocytes the CBM complex participates in the signal transduction pathway that connects PAMPs recognition to NF-*κ*B activation. Also, together with the evidences shown before, these experiments indicate that ULK2 negatively regulates this pathway, whereas the two psoriasis-linked CARMA2*sh* mutants tested escape such a negative control. This observation is particularly remarkable as TLRs activation is considered to play an important role in the pathophysiology of psoriasis.^28, 29^

### ULK2 promotes lysosomal degradation of BCL10

**Figure 3:**
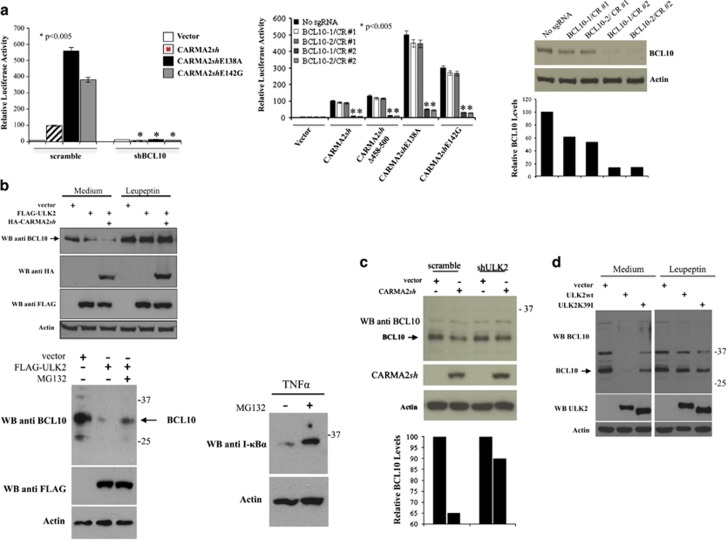
ULK2 promotes degradation of BCL10. (**a**) Left panel: HEK293T cells infected with a lentiviral vector expressing a shRNA targeting BCL10 or a scramble sequence were transiently cotransfected with expression vectors encoding for the indicated polypeptides, together with NF-*κ*B-luciferase and *β*-galactosidase reporter vectors. About 24 h after transfection, cell lysates were prepared and luciferase activity was measured. Data shown represent relative luciferase activity normalized against *β*-galactosidase activity and are representative of at least 10 independent experiments done in triplicate. Data were analyzed by Student's *t*-test, and a *P*-value ⩽0.05, indicated with an *, was considered significant. Right panel: two independent HEK293T lines (BCL10-1/CR#2 and BCL10-2/CR#2) stably harboring Cas9 and a sgRNA targeting BCL10 were used for the NF-*κ*B luciferase assay described before. The cell lines BCL10-1/CR#1 and BCL10-2/CR#1, harboring an unproductive BCL10 sgRNA, were used as a control. Lower panel: immunoblot and densitometric analysis of BCL10 expression level in BCL10-targeted HEK293T cell lines. (**b**) HEK293T cells were transiently transfected with the indicated expression vectors and after 24 h treated or not with lysosomal (Leupeptin, 10 *μ*M) or proteasomal (MG132, 10 *μ*M) inhibitors 1 h before lysis. Endogenous BCL10 expression was assessed by immunoblot assay. Lower panel: MG132 efficacy was monitored by assessing proteasomal degradation of I*κ*B*α* following TNF*α* stimulation (20 ng/ml for 30 min). (**c–d**) HEK293T cells expressing the indicated shRNAs and/or polypeptides were assessed for endogenous BCL10 expression by western blot assay

**Figure 4:**
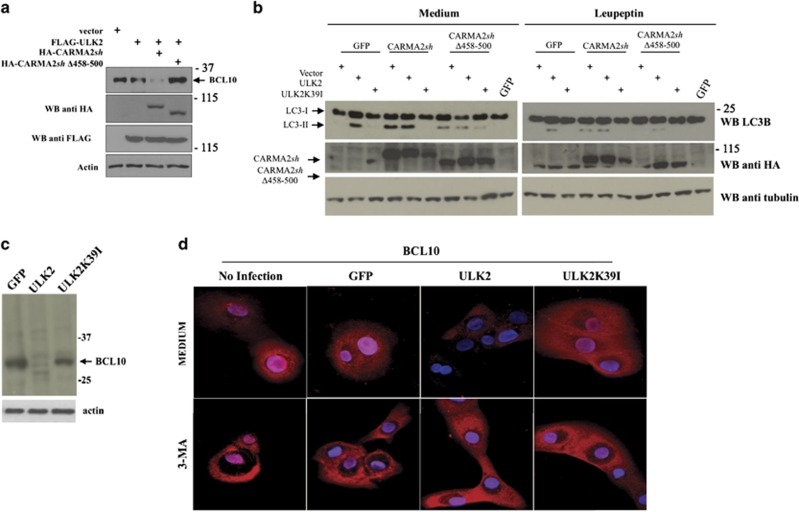
ULK2 promotes degradation of BCL10. (**a**) HEK293T cells were transiently transfected with expression vectors encoding for the indicated polypeptides. About 24 h later, cell lysates were prepared and monitored for endogenous BCL10 expression by immunoblot assay. (**b**) HEK293T cells were transiently transfected with expression vectors encoding for the indicated polypeptides. About 24 h after transfection, cells were left untreated or treated with the lysosome inhibitor leupeptin (10 *μ*M) for 1 h. Cell lysates were then prepared and monitored for conversion of LC3-I to LC3-II by immunoblot assay. (**c**) NHEK were transfected with an expression vector encoding for wt ULK2 or the kinase-dead mutant ULK2K39I. About 16 h later, cell lysates were prepared and BCL10 expression was determined by immunoblot assay. (**d**) Immunofluorescence analysis of BCL10 in NHEK infected with lentiviral vectors expressing the indicated polypeptides and left in medium alone or medium supplemented with the autophagy inhibitor 3-MA (5 mM). Data shown are representative of three independent experiments

The NF-*κ*B-inducing activity of both wt- and psoriasis-linked CARMA2*sh* mutants requires BCL10, as assessed by experiments based on shRNA-mediated depletion of BCL10 (Figure 3a, left panel) and disruption of the BCL10 locus through CRISPR/Cas9/sgRNA technology in HEK293T cells (Figure 3a, right panel). Moreover, in the BCL10-targeted cell lines BCL10-1/CR#2 and BCL10-2/CR#2 re-introduction of a plasmid-encoded BCL10 fully restores the NF-*κ*B-inducing activity of CARMA2*sh* (data not shown). Therefore, we explored the possibility that ULK2 could inhibit the NF-*κ*B-inducing activity of CARMA2*sh* by regulating the expression level of BCL10. Indeed, as shown in Figure 3b, in HEK293T cells expression of CARMA2*sh*, which mimics activation of the CBM complex with consequent NF-*κ*B activation, results in a reduction of endogenous BCL10 protein level, which was further accentuated by co-expressed ULK2. BCL10 reduction was rescued by the lysosomal inhibitor leupeptin, but not by the proteasomal inhibitor MG132 (Figure 3b upper and lower panels). Consistently, the lysosomal inhibitor 3-methyladenine (3-MA) rescued the inhibitory effect of ULK2 on the NF-*κ*B-inducing activity of CARMA2*sh* (Supplementary Figure 2). Reduction of endogenous BCL10 following CARMA2*sh* expression requires the kinase activity of ULK2, as it was not observed when either shRNA targeting ULK2 or ULK2K39I were used (Figures 3c and d). ULK2-mediated reduction of BCL10 requires phosphorylation of CARMA2*sh*, as it was not observed when the mutant CARMA2*sh*Δ458-500 was used (Figure 4a).

Together with the experiments done in the presence of lysosomal inhibitors, examination of LC3 conversion from LC3-I to LC3-II by immunoblot assay further pointed to a autophagic route of BCL10 triggered by wt ULK2, but not ULK2K39I, and promoted by wt CARMA2*sh* but not by CARMA2*sh*Δ458-500 (Figure 4b). Finally, reduction of endogenous BCL10 was also observed in NHEK upon expression of wt ULK2 but not ULK2K39I, and it was rescued by the lysosomal inhibitor 3-MA (Figures 4c and d). Thus, ULK2 negatively regulates the CBM complex signaling, most likely by promoting lysosomal degradation of BCL10.

### Psoriasis-linked CARMA2sh mutants fail to promote BCL10 degradation

**Figure 5:**
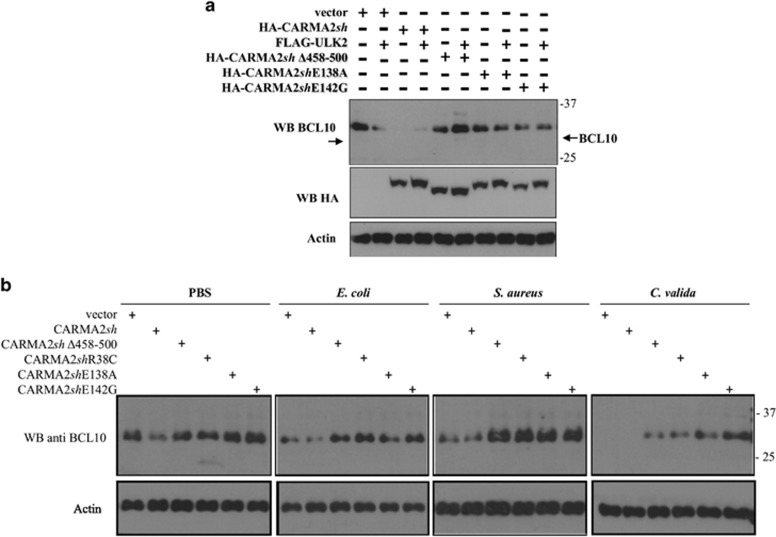
Psoriasis-linked CARMA2*sh* mutants fail to promote BCL10 degradation. (**a**) NHEK cells were transiently transfected with expression vectors encoding for the indicated polypeptides and analyzed for endogenous BCL10 expression by immunoblot assay. (**b**) NHEK infected with retroviral vectors encoding for the indicated polypeptides were exposed to the indicated stimuli for 4 h and the expression level of BCL10 was determined by immunoblot experiments. Data shown are representative of three independent experiments done in triplicate

Next, we investigated for a mechanism that would explain why CARMA2*sh*E138A and CARMA2*sh*E142G mutants elude the ULK2 negative control on NF-*κ*B activation. First of all, we examined whether CARMA2*sh*E138A and CARMA2*sh*E142G mutants are still able to associate to and be phosphorylated by ULK2. The experiments shown in Supplementary Figure 3 indicate that, in transfection experiments, the non-phosphorylatable mutant CARMA2*sh*Δ458-500 and the psoriasis-linked CARMA2*sh*E138A and CARMA2*sh*E142G mutants are all still able to co-immunoprecipitate with ULK2. In addition, as judged by a shift in the SDS/PAGE mobility, both CARMA2*sh*E138A and CARMA2*sh*E142G mutants appear to be phosphorylated by ULK2 (Supplementary Figure 3). Thus, phosphorylation of CARMA2*sh* by ULK2 is required but not sufficient for ULK2-mediated inhibition of the NF-*κ*B-inducing activity of CARMA2*sh.* Thus, we explored the possibility that CARMA2*sh*E138A and CARMA2*sh*E142G might fail to promote degradation of BCL10. Indeed, reduction of endogenous BCL10 protein was not observed when the psoriasis-linked mutants CARMA2*sh*E138A or CARMA2*sh*E142G were expressed with ULK2 in NHEK (Figure 5a).

We then verified the possibility that the inability of CARMA2*sh*E138A and CARMA2*sh*E142G to promote degradation of BCL10 was somehow associated with their higher capacity to activate NF-*κ*B. For this, we tested an additional psoriasis-linked mutant, CARMA2*sh*R38C, which is incapable to activate NF-*κ*B.^6^ However, as shown in Supplementary Figure 4, also CARMA2*sh*R38C is unable to support degradation of BCL10.

Moreover, following exposure to PAMPs, BCL10 degradation could be readily observed in mock-transfected NHEK (Figure 5b), or NHEK-expressing wt CARMA2*sh,* but not in NHEK expressing the mutants CARMA2*sh*Δ458-500, CARMA2*sh*E138A or CARMA2*sh*E142G (Figure 5b). Analysis of BCL10 mRNA expression in NHEK did not reveal any significant change upon ULK2 or CARMA2*sh* isoforms expression (data not shown), confirming that ULK2-mediated reduction of BCL10 occurs at protein level. Altogether, these data indicate that ULK2-mediated lysosomal degradation of BCL10 represents an intrinsic homeostatic mechanism that restricts NF-*κ*B signaling in keratinocytes, and the psoriasis-linked CARMA2*sh* mutants we tested failed to promote such a constraining mechanism.

**Figure 6:**
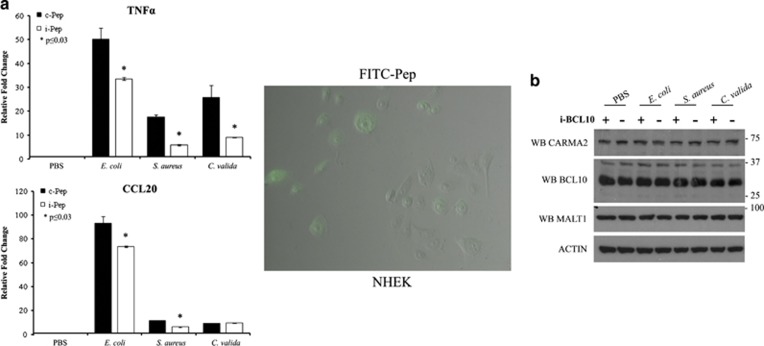
(**a**) BCL10 inhibitory peptide represses NF-*κ*B signaling upon PAMPs recognition in NHEK. NHEK were liposome-delivered with a BCL10 inhibitory peptide (i-BCL10) or a control scrambled peptide (c-BCL10) and exposed to PAMPs for 24 h. Expression level of the indicated NF-*κ*B responsive genes was determined by real-time PCR. Data shown are representative of six independent experiments done in triplicate. Right panel: FITC-conjugated i-BCL10 peptide was used to monitor delivery efficiency (≅50%). (**b**) The expression levels of BCL10, MALT1 and CARMA2*sh* in NHEK left untreated or i-BCL10-delivered were assessed by western blot

Overall, the data shown above indicate that BCL10 is a key regulator of the NF-*κ*B-inducing activity of wt- and psoriasis-associated CARMA2*sh* mutants. Thus, we tested whether liposome-assisted delivery of a BCL10 inhibitory peptide^30^ could reduce NF-*κ*B activation in NHEK exposed to PAMPs. Indeed, the expression level of two monitored NF-*κ*B target genes was partially but significantly diminished when NHEK were treated with a BCL10 inhibitory peptide, but not a control peptide (Figure 6).

## Discussion

In this paper we report the identification of ULK2 as a kinase that promotes phosphorylation of CARMA2*sh,* very likely in the linker region of the protein. This evidence is of particular interest, as it is already known that phosphorylation events occurring in the linker region of CARMA1 control its functional activity. In lymphoid cells, PKC-mediated phosphorylation of the linker region of CARMA1 controls NF-*κ*B activation by triggering a shift from an inactive to an active CARMA1 conformer.^31, 32, 33^ Thus, a CARMA1 mutant in which Ser552 is mutated fails to mediate TCR-induced NF-*κ*B activation in CARMA1-deficient T cells, whereas deletion of the linker results in constitutive, receptor- and PKC-independent NF-*κ*B activation. As a consequence, constitutive activation of NF-*κ*B driven by mutations in the linker region of CARMA1 is considered to be the molecular basis of some B-cell malignancies.^34, 35^ We find now that phosphorylation of the linker region in CARMA2*sh* is somehow also required to trigger the subsequent lysosomal degradation of BCL10, that is, as a signal necessary to terminate CBM complex-mediated activation of NF-*κ*B. Further studies are needed to verify if such a regulatory mechanism applies for CARMA1 as well, as lysosomal degradation of BCL10 has been proposed as an NF-*κ*B signal-off mechanism in activated lymphocytes.^17, 18, 19^ Our prediction would be that the constitutive NF-*κ*B activity resulting from the linker mutants of CARMA1 also derives from failure to degradate BCL10. In fact, as the NF-*κ*B-inducing activity of CARMA1 requires BCL10, either these mutants do not support degradation of BCL10, or, vice versa, they would be unable to produce constitutive NF-*κ*B activation.

We also show that at least three CARMA2*sh* mutants associated with genetic psoriasis fail to promote degradation of BCL10, despite being phosphorylated by ULK2. Thus, our data indicate that although phosphorylation of CARMA2*sh* is a necessary signal to initiate degradation of BCL10, possibly further components must be recruited for selective BCL10 degradation to take place. Much of the information available on ULK2 comes from studies conducted on its close family member ULK1, which represents a central component of the autophagy regulatory complex.^23, 36^ Although derived from studies on ULK1, these data are perfectly consistent with the evidence shown in our work, pointing to an essential role for ULK2 in triggering lysosomal degradation of BCL10 as a mechanism for quenching NF-*κ*B-activating stimuli conveyed through the CBM complex.

We also demonstrated that a CBM complex comprising CARMA2*sh* in human keratinocytes transduces NF-*κ*B signaling upon PAMPs recognition on the cell membrane. Evidence in this direction, at least for BCL10 and MALT1, already exist in the literature;^20, 21, 22^ however, our discovery made in keratinocytes is particularly evocative, either for the historical association between the onset of psoriasis and bacterial infections, either for more recent associations between genetic variants of TLR4, a major player in PAMPs recognition and psoriasis.^37, 38–39^ Also, this observation is consistent with three recent papers published while this work was in preparation, showing that the proteolytic activity of MALT1 is required for CARMA2*sh*-induced activation of NF-*κ*B.^40, 41, 42^

In conclusion, here we have shown that the CBM complex has an essential role in the activation of the NF-*κ*B pathway following human keratinocytes exposure to PAMPs, and that ULK2-mediated degradation of BCL10 represents a mechanism through which this signaling is turned off. Degradation of BCL10 by ULK2 requires phosphorylation of CARMA2*sh*, and is prevented by several psoriasis-linked CARMA2*sh* mutations. Thus, the inflammatory phenotype observed in psoriatic skin may result from unrestrained NF-*κ*B signaling due to deficiency of lysosomal BCL10 degradation. Certainly, our data do not exclude the possibility that in addition to the lysosomal pathway also other posttranscriptional mechanisms may contribute to degradation of BCL10. Indeed, as we show in the present work, targeting of BCL10 may represent a novel approach for the treatment of this debilitating disease.

## Materials and methods

### Two-hybrid screening

The two-hybrid screening was performed using the Matchmaker system (Clontech, Mountain View, CA, USA) as previously described.^43^ Briefly, yeast strain AH109 GAL4^−/−^ was first transformed with pGBKT7 plasmids carrying the CARMA2*sh* cDNA bait fused with DBD of GAL4 using lithium acetate/PEG 3000 procedure. Transformant colonies were selected on synthetic dropout plates lacking tryptophan. Expression of bait fusion proteins was assessed by immunoblot analysis. For library screening, yeast AH109 expressing GAL4DBD-CARMA2*sh* was transformed with a human fetal brain cDNA library cloned in pACT2 vector (Clontech) in fusion with GAL4TAD. About 2 × 10^6^ clones were screened for interaction with GAL4DBD-CARMA2*sh* using selective growth on minimal medium lacking nutrients whose biosynthesis is mediated by genes under control of the GAL4 transcriptional activity.

### Cell culture, plasmids and antibodies

HEK293T and HaCaT cells were obtained from ATCC and cultured in Dulbecco's modified Eagle's medium supplemented with 10% FCS. NHEK were purchased from Lonza (Basel, Switzerland) and cultured according to the provided instructions. HEK293T were transfected by calcium phosphate precipitation; NHEK were transfected using DreamFect Gold Transfection Reagent (OZ Biosciences, Marseille, France) according to the manufacturer's instruction. HaCaT were transfected with Lipofectamine 3000. Retroviral infections were carried out as previously described.^44^ CARMA2*sh* and ULK2 mutants were generated through PCR-mediated methods and confirmed by sequencing. Lentiviral vectors expressing shRNAs targeting ULK2 were a gift from Dr. R Shaw (Addgene plasmid #27634, Cambridge, MA, USA). The list of primers and oligos used for this study is shown in the Supplementary Figure 5.

Sources of antisera and monoclonal antibodies were the following: anti-FLAG, anti-*β*-actin, anti-ULK2, Sigma-Aldrich (St. Louis, MO, USA); anti-CARMA1, Santa Cruz Biotechnology (Dallas, TX, USA). Antisera against BCL10 and CARMA2 were generated in our laboratory and were previously described.^11, 45^

To disrupt *BCL10* gene, a CRISPR/Cas9/single guide (sg) RNA system was designed.^46^ The sg sequences were cloned into LentiCRISPRv2 containing a Cas9 expression cassette (Addgene) and transduced into HEK293T cells. After puromycin (3 *μ*g/ml) selection, cells stably expressing Cas9/BCL10 sgRNA were isolated. The efficiency of BCL10 knockout was determined by immunoblotting.

### Immunoblot analysis and coprecipitation

Cell lysates were made in lysis buffer (150 mM NaCl, 50 mM Tris, pH 7.2, 1% NP40, 2 mM EDTA and a mixture of protease inhibitors). Proteins were separated by SDS-PAGE, transferred onto nitrocellulose membrane and incubated with primary antibodies followed by horseradish peroxidase-conjugated secondary antibodies (Amersham Biosciences, Piscataway, NJ, USA). Blots were developed using the ECL system (Amersham Biosciences). For coimmunoprecipitation experiments, cells were lysed in lysis buffer and immunocomplexes were bound to protein A/G (Roche, Basel, Switzerland), resolved by SDS-PAGE and analyzed by immunoblot assay. All immunoblots were done at least three times using different biological material as sources. Phosphatase Inhibitor Cocktail was purchased from Sigma and used according to the instructions provided.

### Luciferase assay

To assess for NF-*κ*B activation state, HEK293T cells were transfected with the indicated plasmidic DNAs together with pNF-*κ*B-luc (Clontech) in 6-well plates. Twenty-four hours after transfection, luciferase activity was determined with Luciferase Assay System (Promega, Madison, WI, USA). Plasmids expressing RSV-*β*-galactosidase or TK-Renilla were used in transfection mixtures in order to normalize for efficiency of transfection.

### Mixed beads *in vitro* kinase assay

HEK293T cells, separately transfected with plasmids encoding for CARMA2 polypeptides and wild-type or mutant ULK2, were lysed in ice-cold TNTE buffer (20 mM Tris, pH 7.5, 150 mM NaCl, 0,3% (vol/vol) Triton X-100, 5 mM EDTA) containing complete protease inhibitor cocktail (Roche), 25 mM *β*-glycerophosphate, 1 mM sodium orthovanadate, 30 nM okadaic acid, 2 mM sodium pyrophosphate. Lysates cleared by centrifugation were incubated with anti-FLAG monoclonal M2 antibody (Sigma Aldrich) for 1 h at room temperature and then washed three times with TNTE buffer. Immunoprecipitates were further washed once with kinase reaction buffer (KRB) (20 mM HEPES, pH 7.5, 20 mM MgCl_2_, 25 mM *β*-glycerophosphate, 2 mM DTT, 100 *μ*M sodium orthovanadate and 30 nM okadaic acid) and then mixed and incubated at a final volume of 20 *μ*l in KRB containing 20 *μ*M ATP at 30 °C for 30 min. Reactions were stopped with the addition of 3% SDS sample buffer during heating at 65 °C for 5 min. Mixed beads reaction product were resolved by SDS-PAGE and analyzed by immunoblot assay.

### Real-time PCR

Total RNA was isolated from cells or tissues using TRIzol reagent (Invitrogen, Carlsbad, CA, USA). The reverse transcriptase reaction was performed using 1 *μ*g of total RNA in a 20 *μ*l reaction and 1 *μ*l of the resulting cDNA was used in the subsequent amplification step along with 300 nM of each primer. The geometric mean values of *β*-actin and succinyl-CoA synthetase *β*-subunit fragment were used as normalization factors. The relative transcription level was calculated by using the ΔΔCt method. Real-time PCR reactions were performed in triplicate by using the SYBR Green PCR Master Mix (Qiagen, Hilden, Germany) in a 7900HT system (Applied Biosystems, Foster City, CA, USA).

## Figures and Tables

**Table 1:** Interaction of CARMA2*sh* with ULK2 in the yeast two-hybrid assay

**Protein fused to GAL4 domain**		**Yeast growth on selective media**
**DNA binding**	**Activating**	
—	ULK2^805–1036^	−
Vector	ULK2^805–1036^	−
FADD	ULK2^805–1036^	−
CARMA2*sh*	ULK2^805–1036^	+++
CARMA2*sh*	—	−

Yeast AH109 was transformed with CARMA2*sh* fused to the GAL4-activating domain together with the indicated cDNAs fused to GAL4 DNA-binding domain. The cDNA encoding for FADD served as a putative negative control. Interactions were examined by yeast growth on selective media; assays were done for 10 independent transformants. Yeast colonies were scored as positive when a growth developed within 24–36 h; a negative was scored when growth failed to develop within 1 week
