# Supplementary material for: CARMA2sh and ULK2 control pathogen-associated molecular patterns recognition in human keratinocytes: psoriasis-linked CARMA2sh mutants escape ULK2 censorship
Source: Cell Death Dis. 2017 Feb 23;8(2):e2627–. doi: 10.1038/cddis.2017.51 (PMC5386493; doi:10.1038/cddis.2017.51)
Supplement: Supplementary Figure 2 [file cddis201751x2.docx]

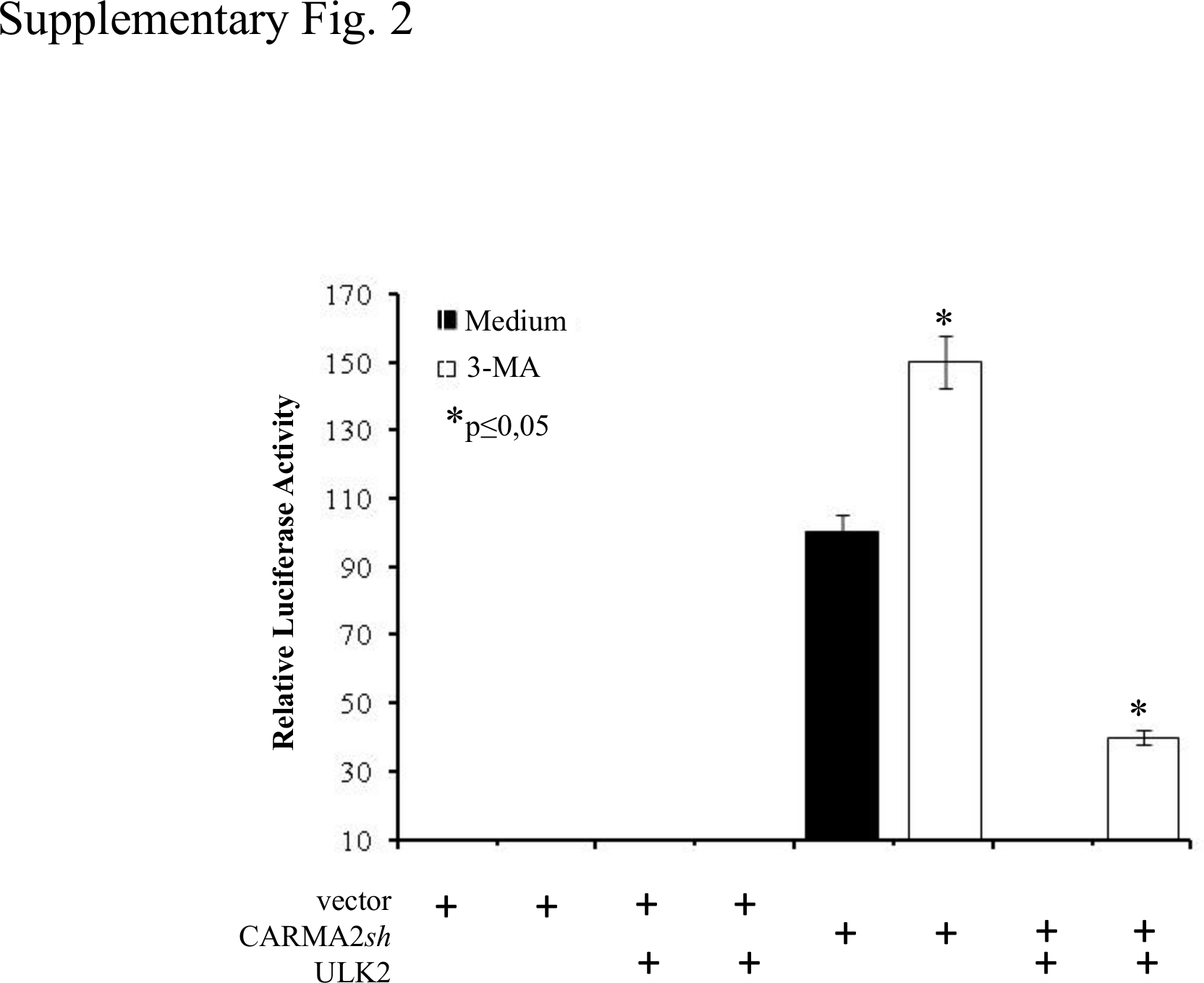


**Supplementary Fig. 2** Lysosomal inhibitor 3-MA rescues the inhibitory effect of ULK2. HEK293T cells were transiently cotransfected with expression vectors encoding for the indicated polypeptides, together with NF-κB-luciferase and β-galactosidase reporter vectors. Cells were left untreated or treated with the lysosomal inhibitor 3-MA (5mM). 24 hrs later, cell lysates were prepared and luciferase activity was measured. Data shown represent relative luciferase activity normalized against β-galactosidase activity and are representative of at least ten independent experiments done in triplicate.
