# Supplementary figures and images for: CARMA2sh and ULK2 control pathogen-associated molecular patterns recognition in human keratinocytes: psoriasis-linked CARMA2sh mutants escape ULK2 censorship
Source: Cell Death Dis. 2017 Feb 23;8(2):e2627–. doi: 10.1038/cddis.2017.51 (PMC5386493; doi:10.1038/cddis.2017.51)

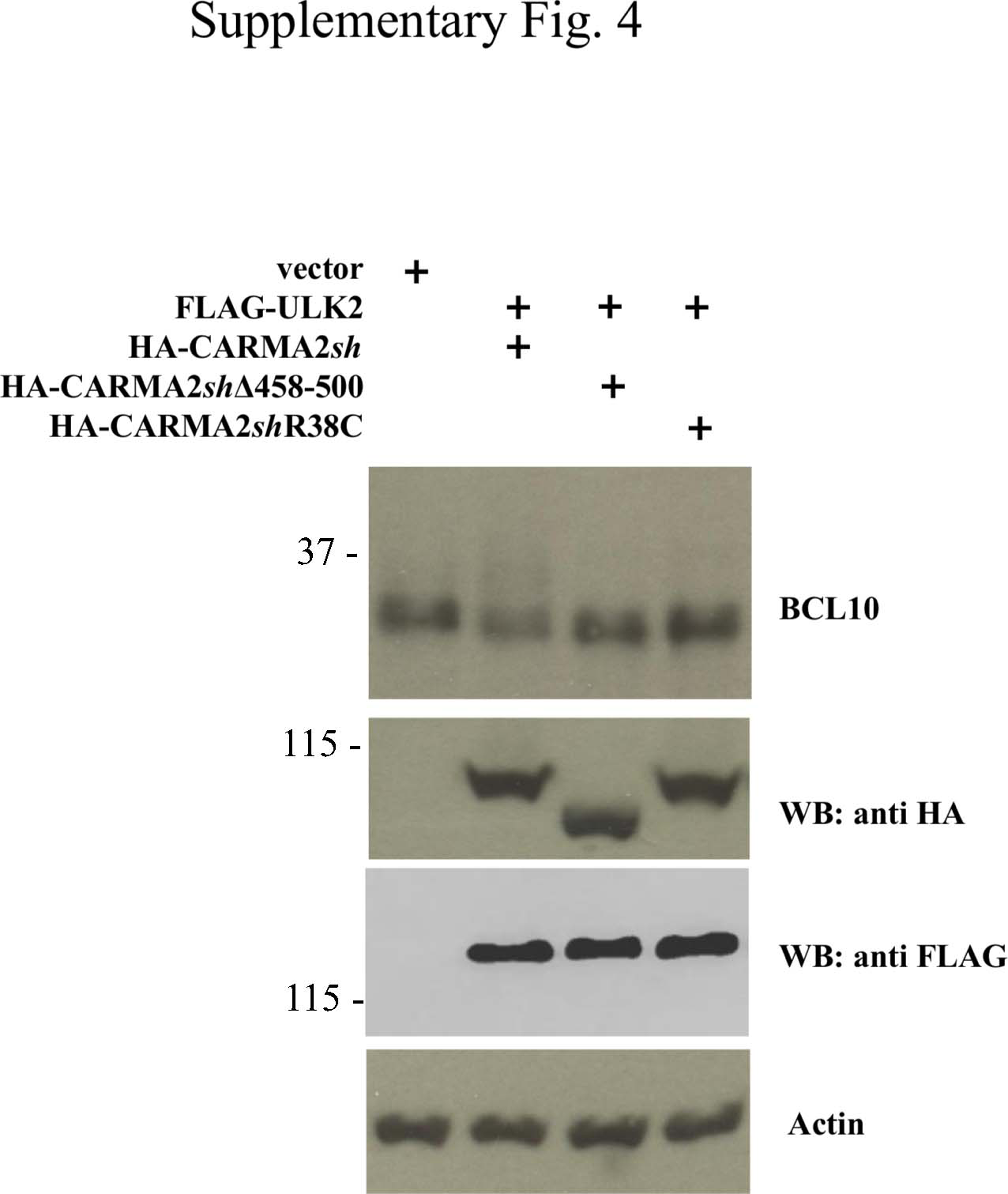

Supplement: Supplementary Figure 4 [file cddis201751x4.tif]
