# Supplementary material for: CARMA2sh and ULK2 control pathogen-associated molecular patterns recognition in human keratinocytes: psoriasis-linked CARMA2sh mutants escape ULK2 censorship
Source: Cell Death Dis. 2017 Feb 23;8(2):e2627–. doi: 10.1038/cddis.2017.51 (PMC5386493; doi:10.1038/cddis.2017.51)
Supplement: Supplementary Figure 5 [file cddis201751x5.docx]

Supplementary Figure 5: List of primers and oligos used for this study.

| **GENE** | **PRIMER SEQUENCE** | |
| --- | --- | --- |
|  | **Forward** | **Reverse** |
| CARMA2 | TCCTAGACACGGCAGACCTT | CCGAGACATCAAGCCTTCCA |
| BCL10 | GGAAGAAGCGCCATCTCCC | CACGTAAATTTTCTAAGGCGTCC |
| MALT1 | AGCCTGTGTCTGCTGAAGTT | GACTGCCTTTGACTCTGGGT |
| ULK2 | GATACGTGCCTTACGGTGCT | TGGCGTAAGGTGTCTGTGTG |
| ACTIN | GAGCACAGAGCCTCGCCTTT | TCATCATCCATGGTGAGCTGG |
| CCL20 | TTGTCTGTGTGCGCAAATCC | CCAACCCCAGCAAGGTTCTT |
| IκBα | ATGCTCAGGAGCCCTGTAATG | TCAGCCCCACACTTCAACAG |
| IL1α | CGGGAAGGTTCTGAAGAAGA | AGGTGCTGACCTAGGCTTGA |
| IL6 | TCAATGAGGAGACTTGCCTG | TGGGTCAGGGGTGGTTATTG |
| IL8 | CACCGGAAGGAACCATCTCA | TGGCAAAACTGCACCTTCACA |
| TNFα | GTTGTAGCAAACCCTCAAGCTG | GAGGTACAGGCCCTCTGATG |
| SDHA | TTGATGCAGTGGTGGTAGC | TTGATTCCTCCCTGTGCTGC |

| **GENE** | **CRISPR** | **SEQUENCE** |
| --- | --- | --- |
| BCL10 | CR#1 | CTCGCCGAATAGATTCAACA |
|  | CR#2 | AGGTTGTTCGTGGCTCCATC |
|  |  |  |

| **Gene** | **Target sequence** | **shRNA oligo sequence** |
| --- | --- | --- |
| CARMA2 | CGTCTCTGTCAACGAGAAGAT | CCGGCGTCTCTGTCAACGAGAAGATCTCGAGATCTTCTCGTTGACAGAGACGTTTTTG |
| BCL10 | CCACCAGATCTACAGTTAGAA | CCGGCCACCAGATCTACAGTTAGAACTCGAGTTCTAACTGTAGATCTGGTGGTTTTTG |
| MALT1 | CCAGAAATCTATTCCAGTATT | CCGGCCAGAAATCTATTCCAGTATTCTCGAGAATACTGGAATAGATTTCTGGTTTTTG |
| ULK2 | GTCAGTGGTATTCGCATCAAA | CCGGGTCAGTGGTATTCGCATCAAACTCGAGTTTGATGCGAATACCACTGACTTTTT |
|  |  |  |
